# Anthrax Lethal Toxin Downregulates Claudin-5 Expression in Human Endothelial Tight Junctions

**DOI:** 10.1371/journal.pone.0062576

**Published:** 2013-04-23

**Authors:** Felice D’Agnillo, Matthew C. Williams, Mahtab Moayeri, Jason M. Warfel

**Affiliations:** 1 Laboratory of Biochemistry and Vascular Biology, Division of Hematology, Center for Biologics Evaluation and Research, Food and Drug Administration, Bethesda, Maryland, United States of America; 2 Laboratory of Respiratory and Special Pathogens, Division of Bacterial, Parasitic and Allergenic Products, Center for Biologics Evaluation and Research, Food and Drug Administration, Bethesda, Maryland, United States of America; 3 Microbial Pathogenesis Section, Laboratory of Parasitic Diseases, National Institute of Allergy and Infectious Diseases, National Institutes of Health, Bethesda, Maryland, United States of America; University Hospital Hamburg-Eppendorf, Germany

## Abstract

Vascular leakage pathologies such as pleural effusion and hemorrhage are hallmarks of anthrax pathogenesis. We previously reported that anthrax lethal toxin (LT), the major virulence factor of anthrax, reduces barrier function in cultured primary human microvascular endothelial cells. Here, we show that LT-induced barrier dysfunction is accompanied by the reduced expression of the endothelial tight junction (TJ) protein claudin-5 but no change in the expression of other TJ components occludin, ZO-1, ZO-2, or the adherens junction (AJ) protein VE-cadherin. The downregulation of claudin-5 correlated temporally and dose-dependently with the reduction of transendothelial electrical resistance. LT-induced loss of claudin-5 was independent of cell death and preceded the appearance of actin stress fibers and altered AJ morphology. Pharmacological inhibition of MEK-1/2, two kinases that are proteolytically inactivated by LT, showed a similar reduction in claudin-5 expression. We found that LT reduced claudin-5 mRNA levels but did not accelerate the rate of claudin-5 degradation. Mice challenged with LT also showed significant reduction in claudin-5 expression. Together, these findings support a possible role for LT disruption of endothelial TJs in the vascular leakage pathologies of anthrax.

## Introduction

Inhalational anthrax is a disease caused by inhaling spores of the gram-positive bacterium *Bacillus anthracis*. Many of the symptoms of systemic inhalational anthrax can be attributed to the action of anthrax toxin, which is made up of three secreted proteins; protective antigen (PA), lethal factor (LF), and edema factor (EF). LF and PA combine to form lethal toxin (LT), while EF combines with PA to form edema toxin [1]. PA binds to the cell surface receptors ANTXR1 and ANTXR2, leading to endocytosis of the enzymatic moieties EF and LF [1], [2]. ANTXR2 appears be the main contributor to lethality *in vivo* with an 11-fold higher affinity for PA than ANTXR1 [3] [4]. Once in the cytosol, EF is a calcium/calmodulin-dependent adenylate cyclase that generates high intracellular concentrations of cAMP [5]. LF is a zinc metalloprotease that inhibits mitogen-activated protein kinase (MAPK) signaling by cleaving all of the upstream MEK proteins except MEK-5 [1]. Serum levels of LF, EF, and PA can exceed microgram per ml quantities during a systemic infection [6] [7] [8].

Systemic anthrax infection is generally associated with profound vascular pathologies including edema, hemorrhage, pleural effusion, and vasculitis in human and animal infections [9] [10] [11] [12]. Pleural effusions and endothelial pathologies have also been observed in animals treated with purified LT [13] [14] [15]. LT increased vascular permeability in a zebrafish model in the absence of endothelial cell death, suggesting that LT may dysregulate endothelial junctions *in vivo* [16]. This latter group also showed that constitutive activation of MEK-1 could counter the vascular effects of LT in this model [17]. Cleavage of MEK-1/2 and altered signaling through the endothelial specific Tie-2 receptor have also been implicated in the permeability changes induced by LT in human endothelial cell culture and *B. anthracis*-infected primates [18]. Combined with the finding that anthrax toxin receptor expression is enriched on the endothelium, these data suggest that targeting of endothelial cells by LT may play a role during systemic anthrax infection [19].

Endothelial barrier function is regulated by adherens junctions (AJs) and tight junctions (TJs), two morphologically distinct structures that are intermingled along the cell-cell junctional cleft [20] [21]. AJs and TJs are each characterized by adhesion molecules that promote homophilic interaction between neighboring cells. The major AJ component, VE-cadherin, is a single-span transmembrane protein that is unique to endothelial cells. TJs are characterized by another endothelial-specific adhesion molecule, claudin-5, which has four membrane-spanning regions. Importantly, the cytoplasmic tails of both VE-cadherin and claudin-5 are linked to the actin cytoskeleton via scaffolding proteins, including the catenin proteins for AJs and zona occludens (ZO)-family proteins for TJs. This interaction with the intracellular cytoskeleton is postulated to provide additional rigidity to the structures and allow for rapid alterations in barrier integrity in response to a variety of stimuli [22] [23].

We previously reported that LT induces cell death-independent endothelial barrier dysfunction in primary human lung microvascular endothelial cells [24]. Morphologically, LT-treated endothelial cells were characterized by the formation of actin stress fibers and alterations in the expression and localization of VE-cadherin [25] [25]. We showed that these alterations were associated with enhanced phosphorylation of myosin light chain (MLC), an actin-associated protein, and dependent upon Rho kinase (ROCK) signaling [25]. However, our previous studies and data presented herein indicate that LT triggers a loss of barrier function *prior to* the observed changes in the actin cytoskeleton and VE-cadherin, suggesting the latter events are secondary consequences of LT action. We therefore hypothesized that LT could be initiating barrier dysfunction by targeting other endothelial junctional complexes. Here, we show that the early loss in barrier function correlates with the LT-mediated inhibition of the transcription and expression of claudin-5 in primary human endothelial cells. Reduced claudin-5 expression is also observed in the livers of LT-treated mice, indicating that effects on the endothelium also occur *in vivo*. Our data suggest a novel mechanism of TJ disruption by LT, which may contribute to the vascular pathogenesis associated with anthrax disease.

## Materials and Methods

### Ethics Statement

The animal study protocol (#LPD8E) was reviewed and approved by the Animal Care and Use Committee of the National Institute of Allergy and Infectious Diseases, National Institutes of Health.

### Reagents

Phosphate-buffered saline (PBS) and Hank’s balanced salt solution with calcium and magnesium (HBSS) were obtained from Invitrogen (Carlsbad, CA). Caspase inhibitors, z-VAD-fmk and DEVD-fmk were purchased from R&D systems (Minneapolis, MN). U0126 (an inhibitor of MEK-1/2), SB230580 (an inhibitor of p38 MAPK), SP600125 (an inhibitor of JNK), MG132, marimastat, E-64, pepstatin A, Y27632, and ML-7 were purchased from EMD Chemicals (Gibbstown, NJ). Cycloheximide, chloroquine, and propidium iodide were purchased from Sigma Chemical Co. (St. Louis, MO). LF, PA, and mutant LF_E687C_ were kindly provided by Dr. Stephen H. Leppla (National Institutes of Health, Bethesda, MD) [26] [27]. The LF used here is a recombinant protein having an N-terminal sequence beginning HMAGG. Toxin proteins were diluted in sterile PBS before cell treatment.

### Antibodies

Mouse IgG1 monoclonal antibodies to ZO-1 (catalog #33-9100), occludin (catalog #33-1500), and claudin-5 (catalog #35-2500) and rabbit polyclonal antibody to claudin-5 (catalog #34-1600) were purchased from Invitrogen (Carlsbad, CA). Antibodies to ZO-2 (catalog #2847), VE-cadherin (catalog #2500), ubiquitin (catalog #3936), PARP (catalog #9542), caspase 3 (catalog #9662), cleaved caspase 3 (catalog #9661), p38 (catalog #9212), JNK1/2 (catalog #9252), ERK1/2 (catalog #9102), HSP27 (catalog #2402), c-Jun (catalog #9165), and the phosphorylated forms of p38 (T180/Y182, catalog #4511), JNK1/2 (T183/Y185, catalog #4668), ERK1/2 (T202/Y204, catalog #4370), c-Jun (S73, catalog #3270), and HSP27 (S82, catalog #2406) were purchased from Cell Signaling Technology (Danvers, MA). Antibodies to MEK-3 (catalog #sc-961), MEK-4 (catalog #sc-837), VE-cadherin (catalog #sc-6458), and tubulin (catalog #sc-9104) were purchased from Santa Cruz Biotechnology (Santa Cruz, CA). Rabbit polyclonal antibody to MEK-1 (catalog #07-641) was obtained from Millipore (Billerica, MA).

### Endothelial Cell Culture and Treatment

Primary human lung microvascular endothelial cells were obtained from Lonza (Walkersville, MD) and cultured as described previously [24]. Cells were grown in phenol red-free MCDB 131 medium (Hyclone, Logan, UT) supplemented with 10 mmol/L L-alanyl-L-glutamine, human epidermal growth factor, hydrocortisone, gentamicin, amphotericin-B, vascular endothelial growth factor, human fetal growth factor-B, recombinant growth factor-1 (R^3^-IGF-1), ascorbic acid, and 5% fetal bovine serum (Lonza). Experimental data were obtained from cells in their third to seventh passages. For experiments, cells were treated with LF and PA individually or in combination (LT). As a negative control, cells were treated with catalytically inactive mutant LF_E687C_ (100 ng/ml) in the presence of 500 ng/ml PA. For caspase inhibitor experiments, confluent monolayers were preincubated with 20 µM z-VAD-fmk or DEVD-fmk for 30 min prior to LT. For MEK and MAPK inhibitor experiments, cell monolayers were treated with 10 µM U0126, 10 µM SP600125, and 20 µM SB208580 prepared in DMSO (final concentration in culture <0.2%).

### Transendothelial Electrical Resistance Measurement

To measure endothelial barrier function, cells were grown to confluence on porous polyester membrane inserts (12 mm diameter, 0.4 µm pore size, Transwell, Corning, Cambridge, MA). The upper and lower compartments contained 0.5 and 1.5 ml of media, respectively. For experimental treatments, toxin proteins or chemical inhibitors were added to the upper compartment. Transendothelial electrical resistance (TEER) was measured using an EVOM volt-ohmmeter connected to a 12-mm Endohm unit (World Precision Instruments, Sarasota, FL). At the indicated time intervals, resistance readings (ohms) were obtained from each insert and multiplied by the membrane area (ohms×cm^2^). The resistance value of an empty culture insert (no cells) was subtracted. Data were collected from duplicate inserts per treatment in each experiment. Values were reported as the percent of basal TEER obtained by dividing the resistance values of each treated monolayer by the resistance value of the control monolayer at each given time point.

### Preparation of Whole Cell Extracts

For whole cell extracts, cells were lysed in ice-cold RIPA buffer (50 mM Tris, 150 mM NaCl, 1% IgePal-630, 0.5% deoxycholate, 1 mM EDTA) containing protease inhibitor mixture (Cocktail Set III, EMD Millipore) and phosphatase inhibitors (Cocktail Set V). Following centrifugation, whole cell supernatants were collected and stored at −80°C. Protein concentration was measured using the BCA assay (Pierce).

### Western Blotting

Reduced samples were run on NuPAGE 4–12% gradient Bis-Tris gels in MOPS SDS running buffer. Proteins were transferred to PVDF membranes, blocked for 1 h in TBS containing 0.1% Tween 20 (TBST) with 5% nonfat dry milk or 3% BSA, and probed with the specific primary Ab followed by HRP-conjugated secondary Ab. Signal was detected on HyperECL film with the ECL Plus chemiluminescence kit (GE Healthcare). For phosphorylated proteins, blots were stripped and reprobed for total protein. Otherwise, blots were stripped and reprobed for tubulin as a loading control. Densitometry analysis was performed using Image J software (National Institutes of Health, Bethesda, MD).

### Immunocytochemistry

Cells were grown to confluence in 24-well dishes and treated as described. At the indicated time interval, cells were fixed in 3.7% paraformaldehyde for 5 min and permeabilized with ice cold methanol for 10 min. After blocking in PBS buffer containing 5% goat serum and 0.25% Triton-X (Tx) for 1 hour at room temperature, monolayers were incubated with a monoclonal anti-claudin-5 antibody in PBS buffer containing 1% BSA and 0.25% Tx overnight at 4°C. Detection was performed using an Alexa Fluor 555-labeled secondary antibody (1∶800 dilution). Nuclei were stained with Hoechst 33342. Photomicrographs were obtained using an Olympus IX71 inverted microscope (Olympus America, Melville, NY). Standardized microscope and software settings were applied during image capture and postprocessing.

### Cell Viability - Calcein AM/Propidium Iodide

Cell viability was assessed by co-staining with calcein AM-propidium iodide. Plasma membrane-permeant calcein AM is cleaved by esterases in live cells to yield cytoplasmic green fluorescence, and membrane-impermeant propidium iodide labels nucleic acids of necrotic cells with red fluorescence. Briefly, cells grown in 24-well dishes were treated as indicated. After an initial wash, adherent monolayers were incubated in medium containing 2 µM calcein AM (Invitrogen) and 3 µM propidium iodide for 20 min at 37°C. Stained cultures were then examined by fluorescence microscopy.

### Real-time PCR

RNA was collected using the RNeasy Mini kit (Qiagen) and converted to cDNA using the TaqMan reverse transcription kit according to the manufacturer’s protocol. Gene expression was analyzed using TaqMan Fast Universal 2× PCR Master Mix (No AmpErase UNG) and TaqMan gene expression assays for *GAPDH* (Hs99999905_m1) and *CLDN5* (Hs01561351_m1) (Applied Biosystems), Reactions were performed in triplicate and run on the Applied Biosystems 7900HT real-time PCR system. Fold gene expression was calculated using the 2^−ΔΔC^T method using *GAPDH* as the reference gene [28].

### Mouse LT Challenge

C57BL/6J mice (12–14 weeks old, female, 22–25 g) were purchased from Jackson Laboratories (Bar Harbor, Maine). Age-matched mice were used for all experiments. Animals were injected intravenously with 50 µg LT (50 µg LF +50 µg PA, prepared in sterile PBS). Mouse livers were harvested and immediately frozen in liquid nitrogen at various times after LT administration. All animal experiments were performed in accordance with guidelines from the NIH and the Animal Welfare Act under protocols approved by the Animal Care and Use Committee of the National Institute of Allergy and Infectious Diseases, National Institutes of Health.

### Western Blot Analyses of Mouse Liver Whole Cell Extracts

For whole cell lysates, liver tissue was homogenized in ice cold RIPA buffer containing protease inhibitor mixture (Cocktail Set III, EMD Millipore) and phosphatase inhibitors (Cocktail Set V). Homogenates were incubated for 30 min on ice and then centrifuged at 15,000 g for 30 min at 4°C. Supernatants were divided into aliquots and stored at −80°C. Protein concentrations were measured by the BCA method. Whole cell extracts were run on NuPAGE 4–12% gradient Bis-Tris gels, transferred to PVDF membranes, and immunoblotted for claudin-5, goat anti-VE-cadherin, MEK-1, PARP, caspase 3. Blots were stripped and probed for tubulin as a loading control. Signal was detected on HyperECL film with the ECL Plus chemiluminescence kit.

### Immunofluorescence Analysis of Claudin-5 in Mouse Liver

Livers were snap frozen in liquid nitrogen, embedded in OCT, and sectioned (HistoServ, Gaithersburg, MD). Frozen sections were fixed in -20°C methanol for 10 min and rinsed in PBS containing 0.05% Tween-20 (PBS-T). Slides were blocked overnight in PBS-T with 0.25% Tx, 10% goat serum and mouse Ig blocking reagent provided in the M.O.M. Basic Kit (Vector Laboratories, Burlingame, CA). Slides were rinsed in PBS-T and incubated for 1 h at room temperature in antibody dilution buffer (PBS-T containing 2% goat serum and M.O.M. protein concentrate as recommended by the manufacturer) containing 0.25% Tx and antibodies to mouse anti-claudin-5 antibody (1∶100). Slides were then rinsed in PBS-T and incubated for 1 h at room temperature in antibody dilution buffer containing Alexa Fluor 555-labeled goat anti-mouse IgG1 (1∶800). Slides were then counterstained with the nuclear stain Hoechst 33342 for 5 min. Separate sections without primary antibody staining were analyzed concurrently to monitor non-specific binding of the secondary antibody. Slides were mounted using Vectashield mounting medium (Vector Labs). Photomicrographs were obtained using an Olympus IX71 inverted microscope (Olympus America, Melville, NY). Standardized microscope and software settings were applied during image capture and post-processing.

### Statistical Analysis

Data are represented as means ± SE for replicate experiments. Statistical analysis was performed by ANOVA with post-hoc Student’s t-test using the JMP (v. 7) software (SAS Institute Inc, Cary, NC). *p*<0.05 was considered statistically significant.

## Results

### LT Reduces Endothelial Barrier Function and Claudin-5 Expression

**Figure 1:**
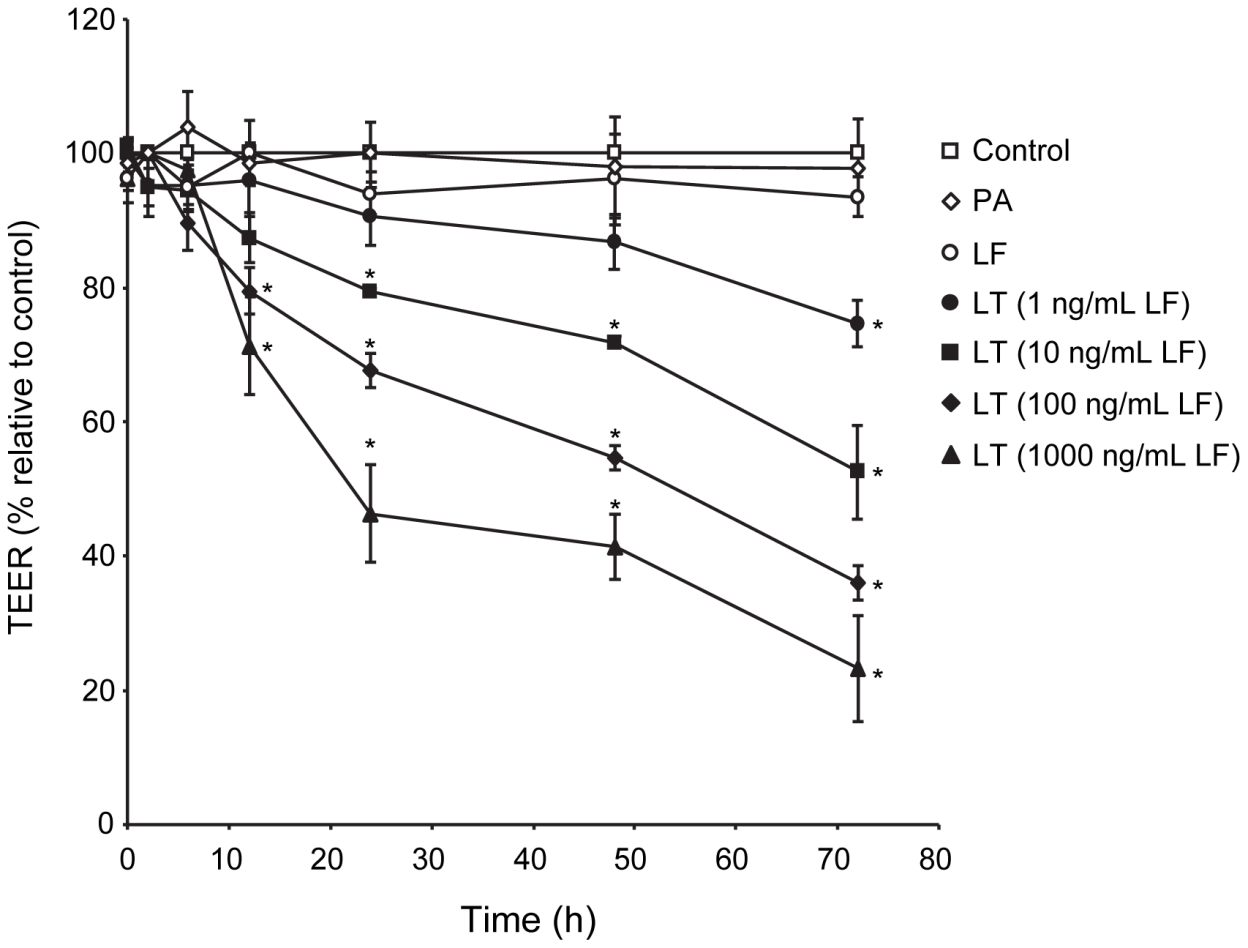
LT induces a time- and dose-dependent reduction in TEER. Cells were treated with medium alone (control), 100 ng/ml LF, 500 ng/ml PA, or the combination of 500 ng/ml PA with increasing concentrations of LF. TEER readings were obtained as described in Materials and Methods. Values were reported as the percentage of basal TEER obtained by dividing the resistance values of each treated monolayer by the resistance value of the control monolayer at each given time point. The means ± SE for a minimum of three independent experiments are shown (n = 3–8). *, *p*<0.05 versus control.

Endothelial barrier function was assessed by measuring transendothelial electrical resistance (TEER) over the course of 72 hours in monolayers treated with LF or PA alone, or the combination of PA with increasing concentrations of LF (LT). LF or PA alone did not alter TEER over the course of 72 hours (Figure 1). LT produced a significant decrease in TEER beginning at 12 hours for 100 ng/ml and 1000 ng/ml, 24 hours for 10 ng/ml, and 72 hours for 1 ng/ml LF. These data support and further extend our previous findings that LT induces a dose- and time-dependent loss in barrier function [24].

**Figure 2:**
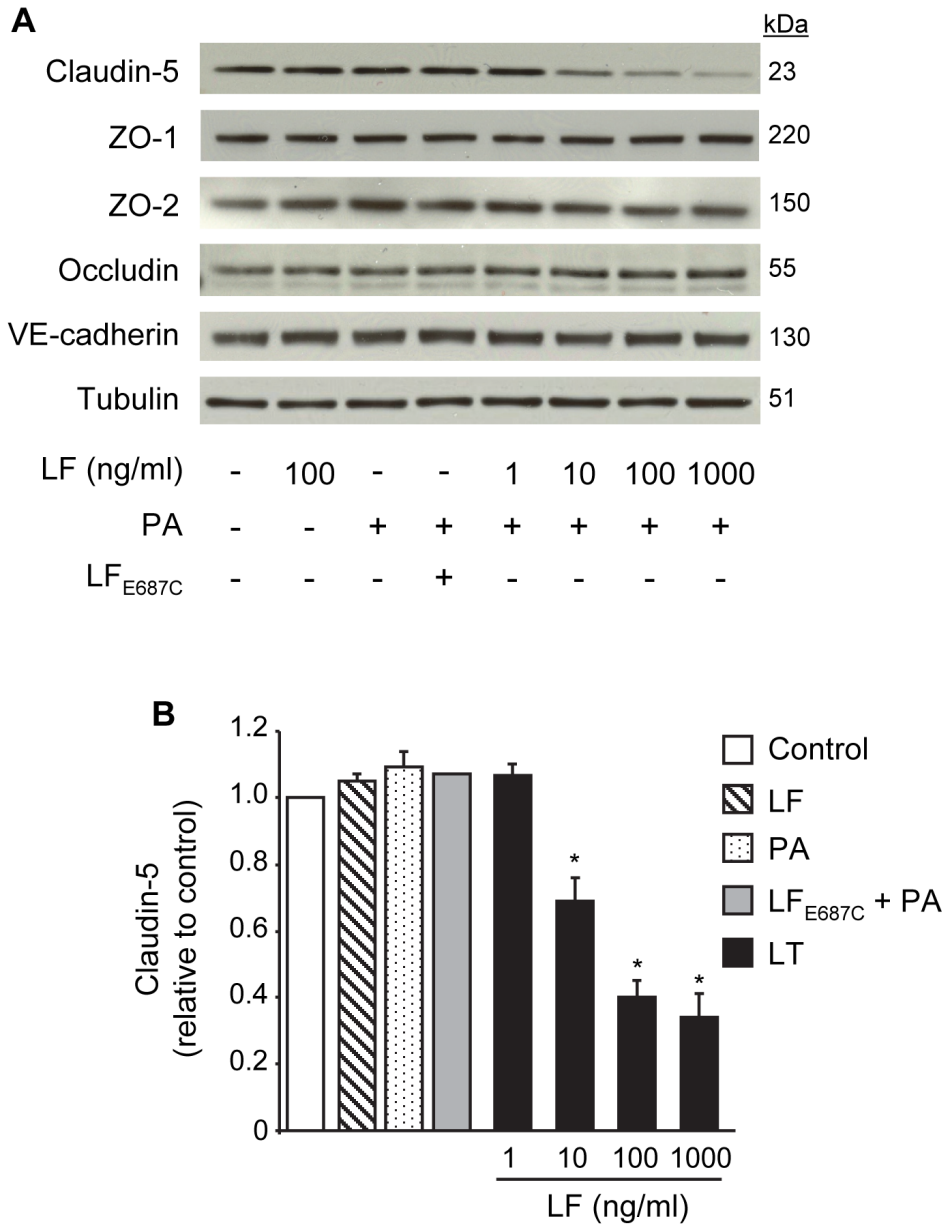
LT reduces claudin-5 expression but does not down-regulate other TJ proteins or VE-cadherin. Cells were treated with medium alone, 100 ng/ml LF, 500 ng/ml PA, inactive mutant LF_E687C_+PA, or PA combined with increasing concentrations of LF for 48 hours. (A) Whole cell lysates were analyzed for claudin-5, ZO-1, ZO-2, occludin, and VE-cadherin. Tubulin served as a loading control. Representative immunoblots of three separate experiments are shown. (B) Claudin-5 expression was normalized to tubulin and presented relative to control. Means ± SE for a minimum of three separate experiments are shown. *, *p*<0.05 versus control.

Next, we examined the effect of LT on the expression of endothelial transmembrane junctional proteins claudin-5, occludin, VE-cadherin, and the scaffolding proteins ZO-1 and ZO-2 by Western blot. LF or PA alone or the combination of PA with the proteolytically inactive mutant LF_E687C_ had no effect on the total expression of any of these proteins after a 48 hour treatment (Figure 2A and B). LT-treated cells showed a LF concentration-dependent reduction in claudin-5 expression but no change in the expression of ZO-1, ZO-2, occludin, or VE-cadherin after 48 hours (Figure 2A and B). In the case of VE-cadherin, these data are consistent with our previous studies that reported a small but significant decrease in VE-cadherin expression at 72 hours but no change at earlier time points [24] [25].

**Figure 3:**
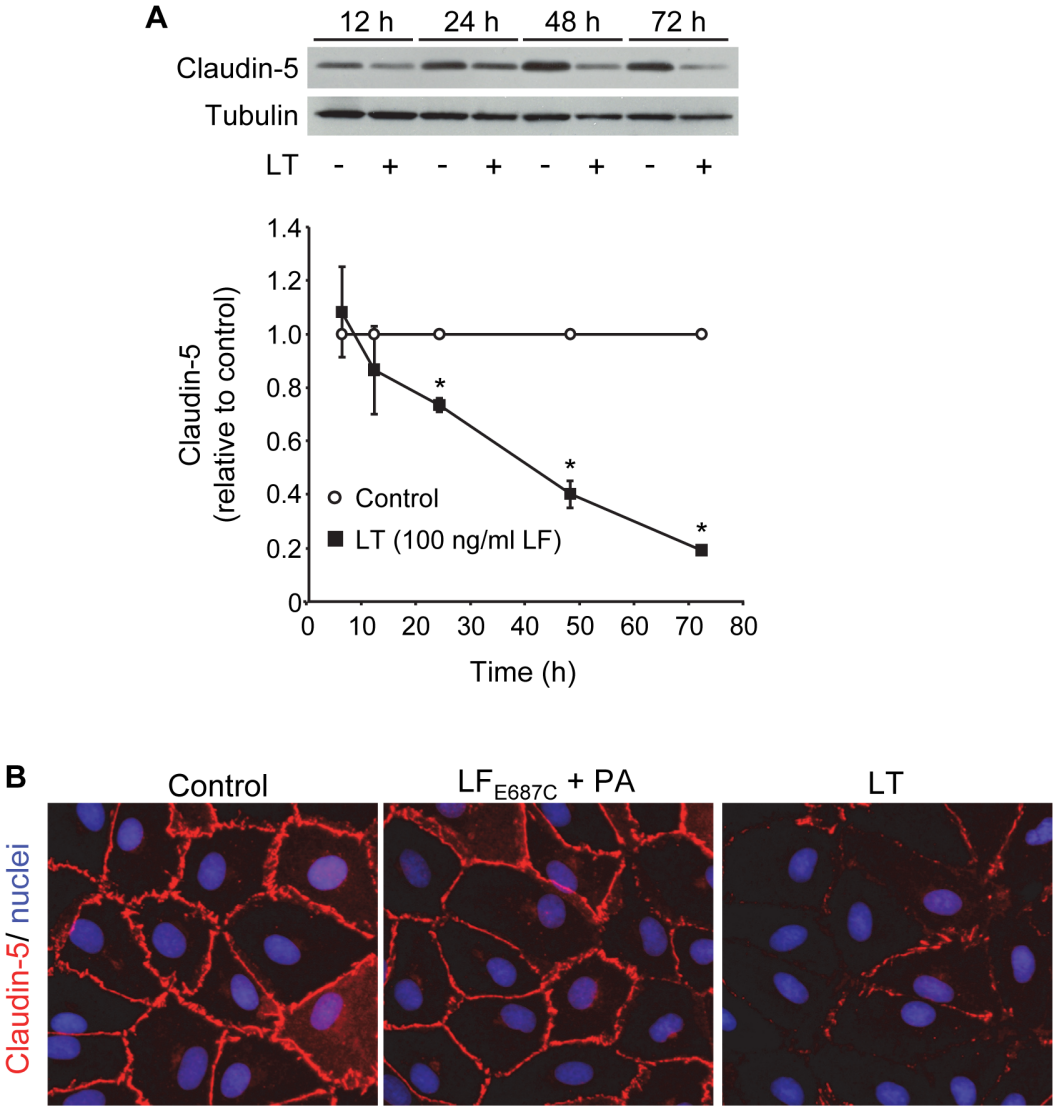
Time-dependent reduction of claudin-5. (A) Cells were treated with medium alone or the combination of 100 ng/ml LF +500 ng/ml PA. Claudin-5 expression was analyzed by Western blot in whole cell lysates collected after the indicated treatment times. Claudin-5 expression was normalized to tubulin and presented relative to control. Means ± SE for a minimum of three separate experiments are shown. *, *p*<0.05 versus control. (B) Immunofluorescence analysis of claudin-5. Cells were treated with medium alone, inactive mutant LT, or LT as described above for 48 hours. Monolayers were stained for claudin-5 (red) as described in Materials and Methods. Nuclei were counterstained with Hoechst 33342 (blue). Images are representative of three separate experiments (400x magnification).

Time-dependent analyses in cells treated with the combination of 100 ng/ml LF and PA showed a small decrease in claudin-5 expression at 12 hours followed by significant reductions at 24 (73±3%, relative to control), 48 (40±5%), and 72 hours (20±1%) (Figure 3A). These data demonstrate a close temporal correlation between the loss of claudin-5 and the reduction of TEER generated with the corresponding 100 ng/ml LF concentration (Figure 1). Consistent with these data, immunofluorescence analyses showed intense staining for claudin-5 at cell-cell junctions in control and inactive toxin-treated cells while inter-endothelial claudin-5 immunoreactivity was sparse or completely absent in LT-treated cells after 48 hours (Figure 3B).

### LT Reduction of Claudin-5 is Cell Death-independent

**Figure 4:**
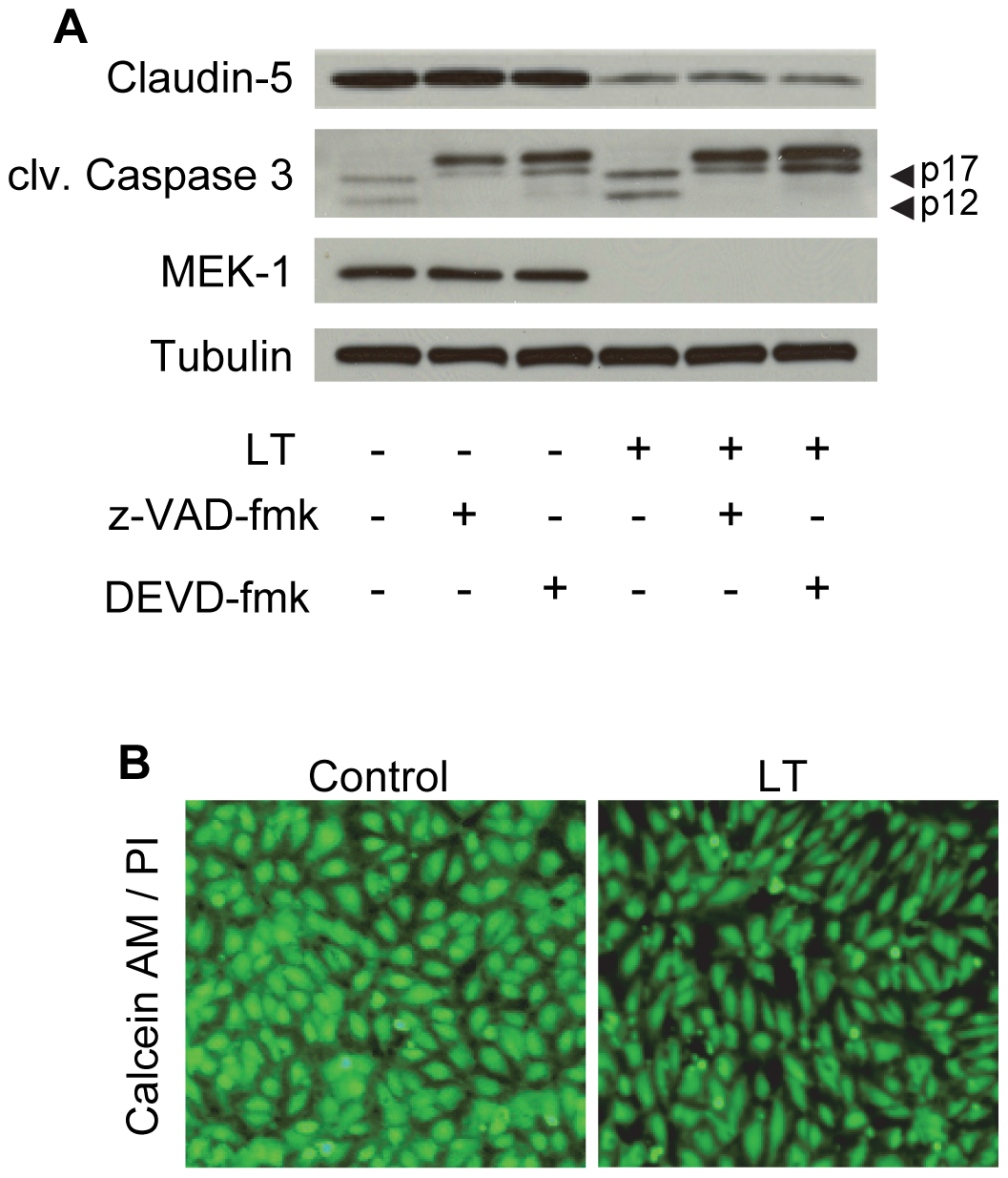
Claudin-5 downregulation is independent of cell death. Cells were pretreated with the caspase inhibitors, z-VAD-fmk (20 µM) or DEVD-fmk (20 µM) for 30 min prior to incubation with LT (100 ng/ml LF +500 ng/ml PA) for 72 hours. (A) Whole cell lysates were analyzed for claudin-5, cleaved caspase 3, and MEK-1 by Western blot. Tubulin served as the loading control. Arrowheads denote the activated p17/p12 fragments of caspase 3. Blots are representative of three separate experiments. (B) Cell viability and necrosis visualized by calcein AM and propidium iodide staining in control- and LT (1000 ng/ml LF +500 ng/ml PA)- treated cells after 72 hours. Images are representative of three separate experiments (100x magnification).

We previously reported that LT-induced barrier dysfunction occurred independently of a minor increase in apoptotic cell death that developed at late time points as measured by FACS analysis [24]. Specifically, we demonstrated that caspase inhibition prevented LT-induced apoptosis and monolayer cell loss but failed to protect against LT-induced TEER reduction and albumin permeability. Consistent with these data, we found that pretreatment with the caspase inhibitors z-VAD-fmk and DEVD-fmk prevented LT cleavage of caspase 3 into its smaller active forms (p12 and p17) but failed to protect against the loss of claudin-5 (Figure 4A). To rule out the possibility that z-VAD-fmk and DEVD-fmk influenced the cellular entry or activity of LT, we showed that these inhibitors had no effect on LT-induced MEK-1 cleavage. To further rule out the potential contribution of cell necrosis, cells were co-stained with calcein AM and propidium iodide (PI) (Figure 4B). After 72 hours, the adherent monolayers in control and LT-treated cultures showed similar calcein signal intensity and no PI-positive staining indicating the absence of necrosis in these cells. Together, these data indicate that LT-induced claudin-5 reduction is independent of cell death. Additional experiments also demonstrated that the reduction of claudin-5 and TEER by LT are not mediated by the activation of ROCK or myosin light chain kinase (MLCK), two major downstream effectors of actin cytoskeletal remodeling (Figure S1).

### Modulation of Claudin-5 Expression by MAPK Pathways

**Figure 5:**
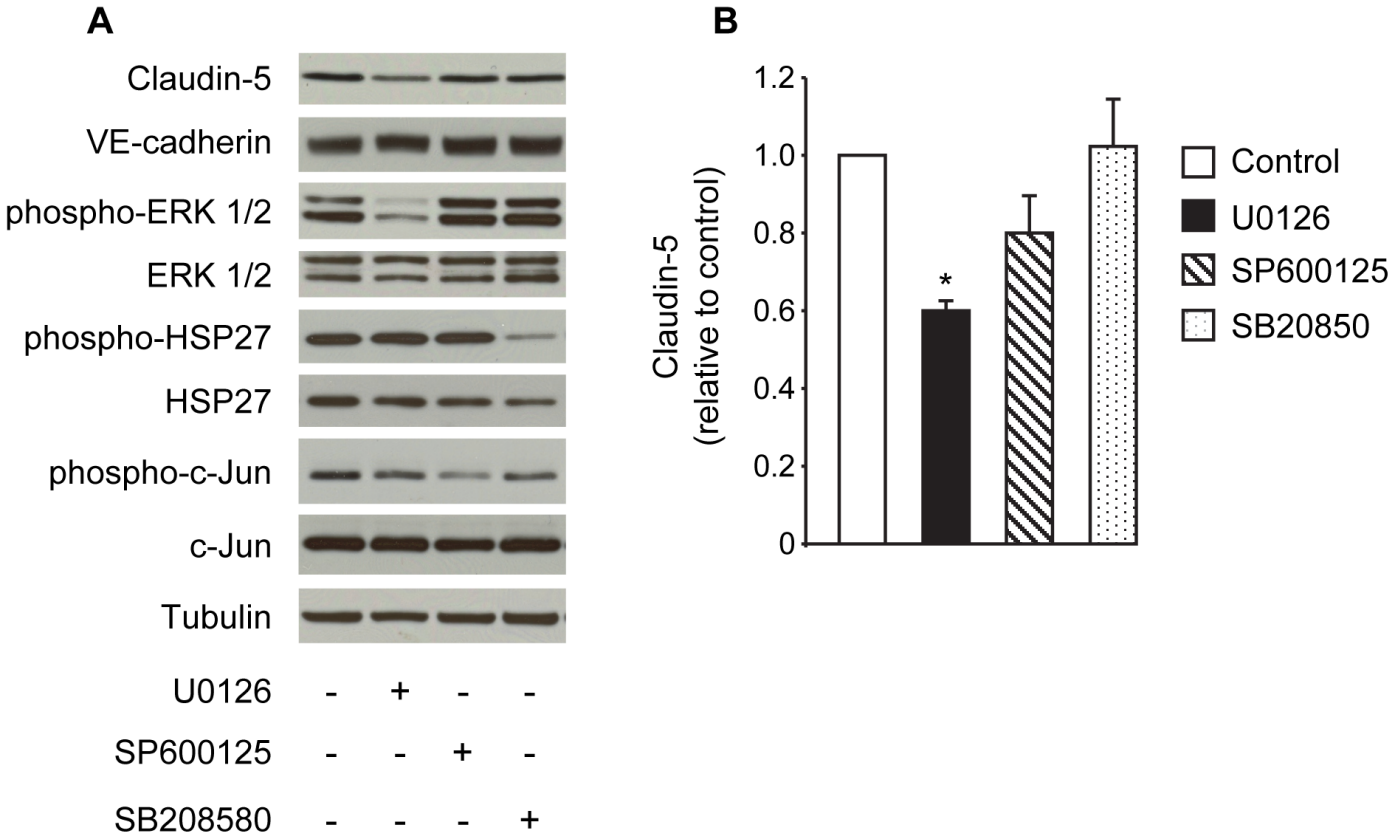
Effect of MEK and MAPK inhibitors on claudin-5 expression. Cells were treated with medium alone, 10 µM U0126, 10 µM SP600125, or 20 µM SB208580 for 48 hours. (A) Whole cell lysates were analyzed for claudin-5, VE-cadherin, and the phosphorylated and total forms of ERK 1/2, HSP27, and c-Jun by Western blot. Tubulin served as the loading control. Representative immunoblots of three separate experiments are shown. (B) Claudin-5 expression was normalized to tubulin and presented relative to control. Means ± SE for a minimum of three separate experiments are shown. *, *p*<0.05 versus control.

Next, we evaluated the inhibitory action of LT on MAPK signaling and its potential involvement in the reduction of claudin-5. Western blot analyses showed that LT produced time- and concentration-dependent cleavage of MEK proteins (MEK-1, -2, and -4) and corresponding decreases in the phosphorylation of ERK1/2, JNK1/2, and p38 (Figure S2). To explore whether any of these individual pathways modulate claudin-5 expression, cells were treated with U0126 (a potent inhibitor of MEK-1/2), SP600125 (a JNK inhibitor), or SB203580 (a p38 inhibitor). Treatment with U0126, but not SP600125 or SB203580, significantly decreased claudin-5 expression after 48 hours (Figure 5A and B). None of the inhibitors altered VE-cadherin levels. The effectiveness of each individual inhibitor was confirmed by monitoring the reduced phosphorylation of ERK 1/2 (a direct target of MEK-1/2), c-Jun (a direct target of JNK), and HSP27 (a downstream target of the p38 pathway) (Figure 5A). These data provide indirect evidence that altered signaling through MEK-1/2 and/or ERK may be a contributing factor in the LT-mediated downregulation of claudin-5.

### LT Downregulates Claudin-5 mRNA Levels but does not Enhance Claudin-5 Degradation

**Figure 6:**
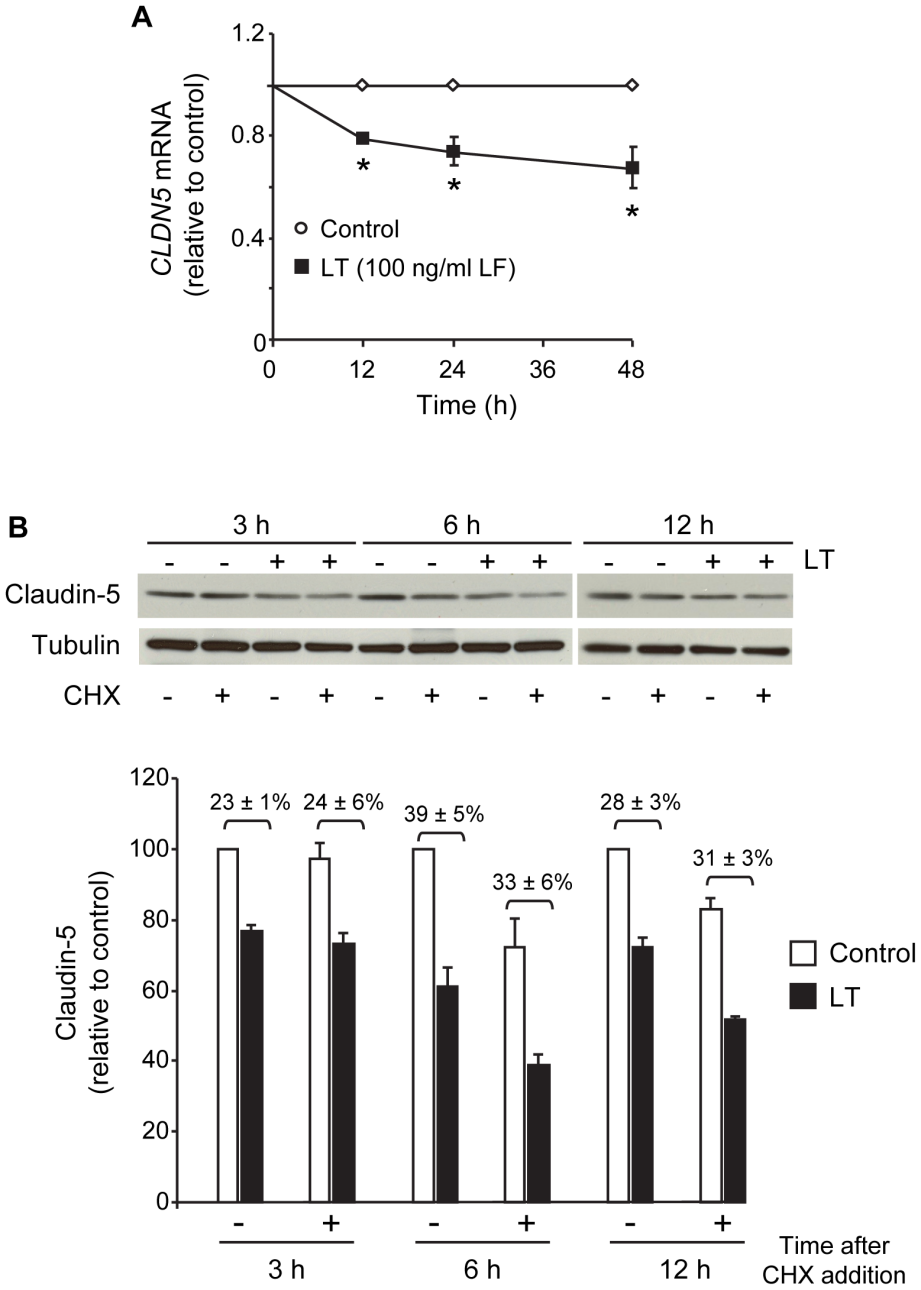
LT reduces claudin-5 mRNA levels but does not accelerate claudin-5 degradation. (A) LT downregulates *CLDN5* gene transcription. Cells were treated with medium alone or the combination of 100 ng/ml +500 ng/ml PA for 12, 24, and 48 hours. RNA was collected and analyzed for *CLDN5* transcript relative to *GAPDH* by real-time PCR. Means ± SE for three separate experiments are shown. *, *p*<0.05 versus control. (B) LT does not accelerate claudin-5 degradation. Cells were treated as indicated above for 18 hours prior to the addition of 5 µg/ml cycloheximide (CHX). Whole cell lysates were collected 3, 6, and 12 hours after CHX addition and analyzed for claudin-5 by Western blot. Claudin-5 expression was presented relative to control without CHX. Means ± SE for a minimum of three separate experiments are shown.

To further investigate the mechanism underlying LT downregulation of claudin-5, we examined the effect of LT on claudin-5 expression at the transcriptional and post-translational level. Real-time quantitative PCR analyses showed that LT reduced *CLDN5* mRNA levels at 12, 24, and 48 hours suggesting that claudin-5 downregulation is at least partly attributed to decreased transcription and/or stability of *CLDN5* mRNA (Figure 6A).

To examine whether LT accelerated the degradation of claudin-5, we performed experiments with the protein synthesis inhibitor cycloheximide (CHX). Cells were incubated with medium or LT for 18 hours and then treated with or without CHX for an additional 3, 6, and 12 hours. Whole cell lysates were collected at each time interval post-CHX and analyzed for claudin-5 by Western blot. At each time interval, CHX had no significant effect on the magnitude of claudin-5 decrease between control and LT-treated cells suggesting that LT does not accelerate claudin-5 degradation (Figure 6B).

**Figure 7:**
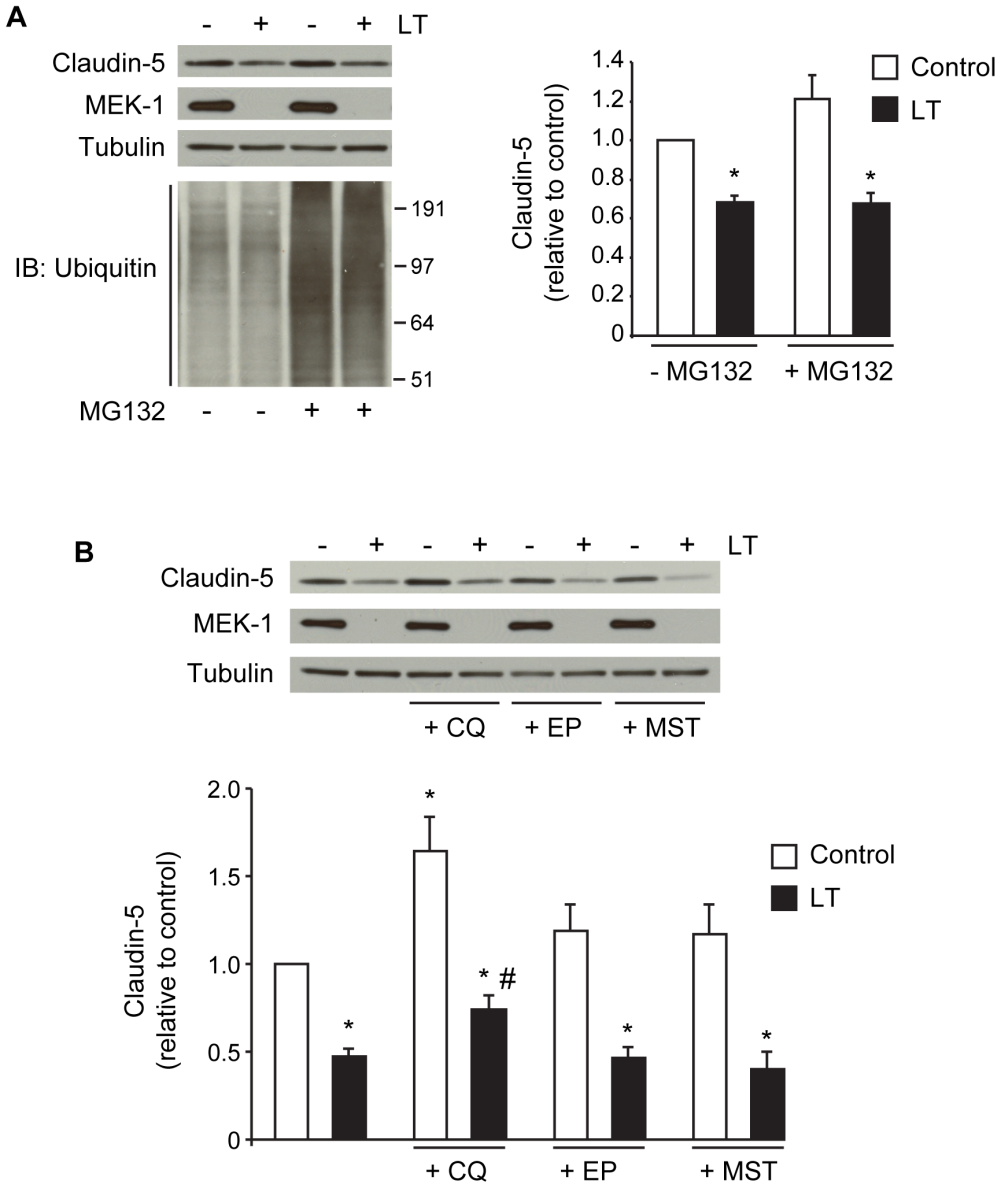
Claudin-5 expression and inhibitors of proteosome, lysosome, and matrix metalloproteinases (MMPs). (A) Cells were treated with medium alone or the combination of 100 ng/ml +500 ng/ml PA for 18 hours prior to the addition of the proteosome inhibitor MG132 (1 µM) for an additional 12 hours. Whole cell lysates were analyzed for claudin-5, MEK-1, and the accumulation of ubiquitinated proteins by Western blot. Representative immunoblots of three separate experiments are shown. Claudin-5 expression was normalized to tubulin and presented relative to control. Means ± SE for a minimum of three separate experiments are shown. (B) Cells were treated as indicated above for 18 hours prior to the addition of lysosome inhibitors, chloroquine (CQ, 20 µM) or E-64 (10 µg/ml) plus pepstatin A (10 µg/ml) (EP), or the broad spectrum MMP inhibitor marimastat (MST, 100 µM) for an additional 24 hours. Whole cell lysates were analyzed for claudin-5 and MEK-1 by Western blot. Claudin-5 expression was normalized to tubulin and presented relative to control. Means ± SE for a minimum of three separate experiments are shown. *, *p*<0.05 versus control, #, *p*<0.05 versus LT alone.

To further support these data, we investigated degradation pathways that have been previously linked to the proteolytic processing of claudins, including the proteosome, lysosome, and matrix metalloproteinases (MMPs) [29] [30] [31]. To examine the possibility that LT enhances proteosomal degradation of claudin-5, cells were incubated with medium or LT for 18 hours and then treated with or without 1 µM of the proteosome inhibitor MG132 for an additional 12 hours. MG132 failed to rescue claudin-5 levels in LT-treated cells (Figure 7A). To confirm the effectiveness of MG132, we monitored the accumulation of ubiquitinated proteins in MG132-treated cells by Western blot. Control and LT-treated cells showed similar baseline levels of ubiquitinated proteins and exhibited similar increases in ubiquitinated proteins in response to MG132. These data are consistent with our previous finding that LT does not enhance or repress proteosome activity in human endothelial cells [32].

To investigate the involvement of the lysosomal pathway, cells were incubated with medium or LT for 18 hours, and then treated with or without the lysosomotropic agent chloroquine (CQ) for an additional 24 hours. In control cells, CQ significantly increased claudin-5 levels suggesting a role for the lysosome in the normal processing of claudin-5 (Figure 7B). CQ partially inhibited the reduction of claudin-5 in LT-treated cells when compared to LT-treated cells without inhibitor. However, the ratio of claudin-5 expression between control and LT-treated cells was about 50% with or without CQ, suggesting this apparent protection is likely due to the inhibitory effect of CQ on normal claudin-5 turnover. To further examine the involvement of the lysosomal pathway, cells were treated with the combination of E-64, an inhibitor of cysteine proteases, and pepstatin A, an inhibitor of aspartic proteases (EP) [33] [34]. Treatment with EP partially increased claudin-5 in control cells but did not inhibit the loss of claudin-5 in LT-treated cells suggesting that LT does not enhance lysosomal degradation of claudin-5 (Figure 7B). Similarly, treatment with marimastat (MST), a broad-spectrum MMP inhibitor, slightly increased claudin-5 in control cells but failed to rescue claudin-5 levels in LT-treated cells (Figure 7B). Together, these data suggest that LT downregulation of claudin-5 likely involves decreased claudin-5 synthesis and not enhanced degradation.

### Reduced Claudin-5 Expression in Livers of LT-challenged Mice

**Figure 8:**
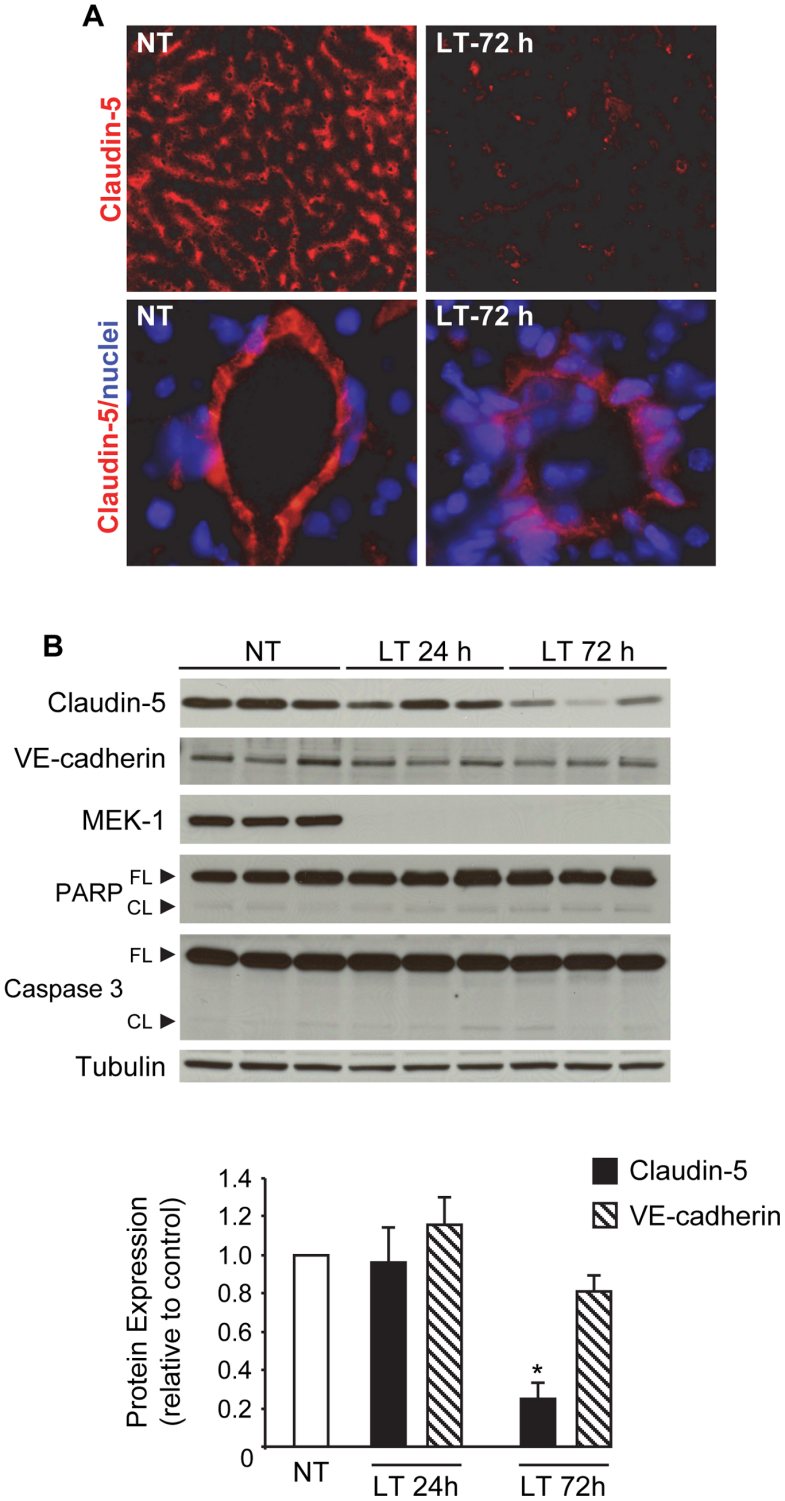
LT reduces claudin-5 expression in mouse liver. Mice were injected with PBS (non-treated, NT) or LT (50 µg LF +50 µg PA). (A) Claudin-5 immunofluorescence analysis in frozen liver sections from NT and LT-72 h mice as described in Materials and Methods. Reduced sinusoidal claudin-5 staining observed in LT-treated mice compared to NT mice (top panels, 200x magnification). Diffused claudin-5 staining also observed in larger hepatic blood vessels in LT-treated mice (bottom panels, 600x magnification). Nuclei were counterstained with Hoechst 33342 (blue). (B) Western blot analyses of claudin-5, VE-cadherin, PARP (full length and cleaved), and caspase 3 (full length and cleaved) in liver whole cell extracts collected from NT and LT mice after 24 and 72 hours. Representative immunoblots of three different animals per group are shown. Claudin-5 or VE-cadherin expression was normalized to tubulin and presented relative to control. Means ± SE for a minimum of three separate animals are shown. *, *p*<0.05 versus control.

To determine whether LT disrupts claudin-5 *in vivo*, we injected mice with LT and analyzed claudin-5 expression in mouse livers by immunofluorescence and Western blot. Previous studies have reported that mice treated with LT develop vascular insufficiency with extensive tissue injury particularly in the liver, spleen, and bone marrow [13] [36] [36]. In PBS-treated mice, claudin-5 expression was detectable in portal veins, hepatic arteries, and sinusoids but absent in central veins. In LT-treated mice, claudin-5 immunoreactivity was significantly reduced in sinusoidal endothelial cells after 72 hours (Figure 8A). Similarly, in larger blood vessels, total claudin-5 immunoreactivity appeared less intense and more diffuse in LT-treated mice compared to PBS-treated controls.

Western blot analyses of liver whole cell lysates showed no significant change in claudin-5 expression 24 hours after LT injection but a greater than 75% reduction after 72 hours (Figure 8B). Comparatively, the expression of VE-cadherin was decreased by 20% relative to control after 72 hours. Minimal cleavage of PARP and caspase 3, markers of apoptosis, was observed in LT-treated mice suggesting minimal apoptotic cell loss in the liver. LT-treated mice showed significant MEK-1 cleavage confirming LT activity in the liver. Taken together, these data support the idea that endothelial claudin-5 may be an important target of LT action *in vivo*.

## Discussion

Growing evidence points to the important role of endothelial dysfunction in anthrax pathogenesis. Animals treated with purified LT succumb to vascular collapse suggesting that the targeting of endothelium by LT may contribute to the pathophysiology of anthrax [13] [14] [15] [36]. We and others have shown that LT induces endothelial barrier dysfunction in a cell death-independent manner [16] [24]. Here, we show that LT-induced loss of barrier function, as measured by TEER, correlates temporally and dose-dependently with reduced claudin-5 levels in endothelial TJs. The loss of TEER and claudin-5 also preceded the formation of actin stress fibers, inter-endothelial gaps, and AJ disorganization that primarily develop at later times (≥72 hours) [24] [25]. Reduced claudin-5 expression was also observed *in vivo*. Claudin-5 levels were reduced by >75% in the livers of LT-treated mice compared to a 20% decrease in VE-cadherin, which correlates well with our *in vitro* observations. Together, these data support the idea that TJ disruption may be an important event underlying the initial stages of LT-induced barrier dysfunction, which in turn may contribute to *in vivo* vascular insufficiency during infection.

We examined several potential mechanisms that could account for the LT-mediated reduction of claudin-5. With regard to the possible involvement of LT-induced cytotoxicity, we showed that the loss of claudin-5 was independent of caspase activation and apoptotic cell loss, consistent with our previous findings that LT induces cell death-independent barrier dysfunction [24]. Given the stabilizing role of the actin cytoskeleton on endothelial junctions, we also considered the possible involvement of ROCK and MLCK, two major downstream regulators of actin remodeling [22] [23] [37]. ROCK has also been shown to regulate TJ integrity by direct phosphorylation of claudin-5 and occludin [38]. We previously showed that inhibitors of ROCK blocked LT-induced MLC phosphorylation, stress fiber formation, and inter-endothelial gap formation [25]. In the present study, however, we found that the downregulation of claudin-5 was independent of ROCK and MLCK activation. These data combined with the early timing of the claudin-5 reduction relative to actin stress fiber formation, supports the interpretation that actin remodeling does not trigger the loss of claudin-5. Another possibility was that LT increased the post-translational degradation of claudin-5. However, experiments with the protein synthesis inhibitor cycloheximide demonstrated that LT does not enhance claudin-5 degradation. In support of this interpretation, we found that inhibitors of the proteosome, lysosome, and MMPs did not rescue claudin-5 levels in LT-treated cells. In agreement with these observations, immunofluorescence analyses showed that there was no detectable difference in the levels of claudin-5 localized in the cytoplasm in control and LT-treated cells suggesting that LT did not enhance the internalization of claudin-5 [39].

We found that the LT-mediated loss of claudin-5 protein correlated with the downregulation of claudin-5 mRNA *in vitro*. One possible explanation for these results is that LT reduces the post-transcriptional stability of claudin-5 mRNA. A previous study reported a destabilizing effect of LT on IL-8 mRNA, although we found that LT had no effect on the stability of VCAM-1 and IRF-1 transcripts in endothelial cells [40] [41]. A second hypothesis is that LT inhibits transcription of claudin-5. We previously reported that LT alters the transcription of several genes involved in endothelial inflammation by increasing or decreasing the activity of the transcription factors NF-κB, IRF-1, and AP-1 via MAPK-dependent and independent processes [32] [41]. While the claudin-5 gene is constitutively expressed by endothelial cells, its transcription can be modulated under certain growth conditions, inflammation, and hypoxia [42] [43] [44]. The claudin-5 promoter is regulated by a number of different transcription factors that can repress (e.g. FOXO1, beta-catenin) or activate transcription (e.g. SOX18) [42] [43] [45]. ETS-related gene (ERG), a major transcriptional regulator of endothelial biology, also controls claudin-5 expression [46] [47] [48]. In preliminary experiments, we found that LT reduces the levels of ERG in the nucleus in both endothelial cells and the livers of LT-injected mice suggesting a possible mechanism for the reduced claudin-5 expression (data not shown). Of potential relevance to LT, several ETS-family transcription factors are regulated through phosphorylation by MAPKs. Phosphorylation of ETS factors has been shown to alter their transactivation potential, DNA binding activity, nuclear localization, and interaction with coregulatory factors [49] [50] [51]. While MAPK-mediated phosphorylation of ERG has not yet been reported, this factor does contain a conserved amino-terminal domain referred to as the Pointed Domain that is typically found in ETS family members that are phosphorylated by MAPKs [51]. In the present study, we showed that the MEK-1/2 inhibitor U0126 decreased claudin-5 expression while inhibitors of JNK and p38 had no significant effect. These observations are in agreement with recent studies that identified a protective role for MEK-1 against LT-induced permeability changes [17] [18]. However, our data must be interpreted with caution because these chemical inhibitors have a limited ability to fully simulate the cellular effects of LT whether used individually or in combination. Further studies are underway in our laboratory to understand the mechanisms by which LT and its action on the MAPKs impacts ERG function and its possible association with claudin-5 downregulation.

We found that the LT-mediated loss of claudin-5 protein correlated with the downregulation of claudin-5 mRNA *in vitro*. One possible explanation for these results is that LT reduces the post-transcriptional stability of claudin-5 mRNA. A previous study reported a destabilizing effect of LT on IL-8 mRNA, although we found that LT had no effect on the stability of VCAM-1 and IRF-1 transcripts in endothelial cells [40] [41]. A second hypothesis is that LT inhibits transcription of claudin-5. We previously reported that LT alters the transcription of several genes involved in endothelial inflammation by increasing or decreasing the activity of the transcription factors NF-κB, IRF-1, and AP-1 via MAPK-dependent and independent processes [32] [41]. While the claudin-5 gene is constitutively expressed by endothelial cells, its transcription can be modulated under certain growth conditions, inflammation, and hypoxia [42] [43] [44]. The claudin-5 promoter is regulated by a number of different transcription factors that can repress (e.g. FOXO1, beta-catenin) or activate transcription (e.g. SOX18) [42] [43] [45]. ETS-related gene (ERG), a major transcriptional regulator of endothelial biology, also controls claudin-5 expression [46] [47] [48]. In preliminary experiments, we found that LT reduces the levels of ERG in the nucleus in both endothelial cells and the livers of LT-injected mice suggesting a possible mechanism for the reduced claudin-5 expression (data not shown). Of potential relevance to LT, several ETS-family transcription factors are regulated through phosphorylation by MAPKs. Phosphorylation of ETS factors has been shown to alter their transactivation potential, DNA binding activity, nuclear localization, and interaction with coregulatory factors [49] [49,50] [49]. While MAPK-mediated phosphorylation of ERG has not yet been reported, this factor does contain a conserved amino-terminal domain referred to as the Pointed Domain that is typically found in ETS family members that are phosphorylated by MAPKs [51]. In the present study, we showed that the MEK-1/2 inhibitor U0126 decreased claudin-5 expression while inhibitors of JNK and p38 had no significant effect. These observations are in agreement with recent studies that identified a protective role for MEK-1 against LT-induced permeability changes [17] [18]. However, our data must be interpreted with caution because these chemical inhibitors have a limited ability to fully simulate the cellular effects of LT whether used individually or in combination. Further studies are underway in our laboratory to understand the mechanisms by which LT and its action on the MAPKs impacts ERG function and its possible association with claudin-5 downregulation.

We present data here that LT downregulation of claudin-5 may be a mechanism for TJ disruption that contributes to LT-induced barrier dysfunction. Interestingly, the idea that LT targets TJs, which primarily regulate the passage of fluid and small molecules, appears to be consistent with the observations that LT-challenged mice develop edema and pleural effusion but not the major hemorrhages observed in experimental and clinical *B. anthracis* infections [9] [11] [13] [15] [52]. It will be important to understand the overall pathogenic relevance of the present findings in the context of an actual anthrax infection where a number of additional factors may converge to affect the severity of vascular dysfunction including aberrant inflammatory processes and the potential contribution of other *B. anthracis*-related toxins [53] [54] [55]. For example, others have shown that LT and ET synergistically block cadherin localization to adherens junctions in endothelial cells by inhibiting endocytic recycling pathways [56]. Mechanistic insights into how anthrax toxin disrupts the endothelial barrier may facilitate the discovery of vascular-directed therapies capable of slowing and/or reversing the severe vascular pathologies of anthrax.

## Supporting Information

Figure S1 **Claudin-5 downregulation is independent of actin cytoskeleton modulators, ROCK and MLCK.** (A) Cells were pretreated with the ROCK inhibitor Y-27632 (5 µM) or the MLCK inhibitor ML-7 (20 µM) for 30 minutes prior to LT (100 ng/ml LF +500 ng/ml PA). Whole cell lysates were collected after 72 hours and analyzed for claudin-5 and MEK-1 by Western blot. Tubulin served as the loading control. Representative immunoblots of three separate experiments are shown. (B) Cells were grown on porous membrane inserts and pretreated with inhibitors prior to LT as indicated above. TEER readings at 72 hours were reported as the percentage of basal TEER obtained by dividing the resistance values of each treated monolayer by the resistance value of the control monolayer. The means ± SE for a minimum of three independent experiments are shown. *, *p*<0.05 versus control.
(TIF)

Figure S2 **LT cleaves MEK proteins and inhibits MAPK phosphorylation.** Cells were treated with medium alone, LF (100 ng/ml), PA (500 ng/ml), or the combination of PA with increasing concentrations of LF for 6, 24, and 48 hours. Whole cell lysates were analyzed by Western blot for MEK-1, MEK-3, MEK-4, and the phosphorylated and total forms of ERK 1/2, JNK 1/2, and p38. Graphs represent the densitometry analyses of phospho-ERK 1/2, JNK 1/2, and p38 as a function of LF concentration at 48 hours. Phospho-MAPK expression was normalized to tubulin and presented relative to control. Means ± SE for a minimum of three separate experiments are shown. *, *p*<0.05 versus control.
(TIF)
