# Supplementary figures and images for: Anthrax Lethal Toxin Downregulates Claudin-5 Expression in Human Endothelial Tight Junctions
Source: PLoS One. 2013 Apr 23;8(4):e62576. doi: 10.1371/journal.pone.0062576 (PMC3633853; doi:10.1371/journal.pone.0062576)

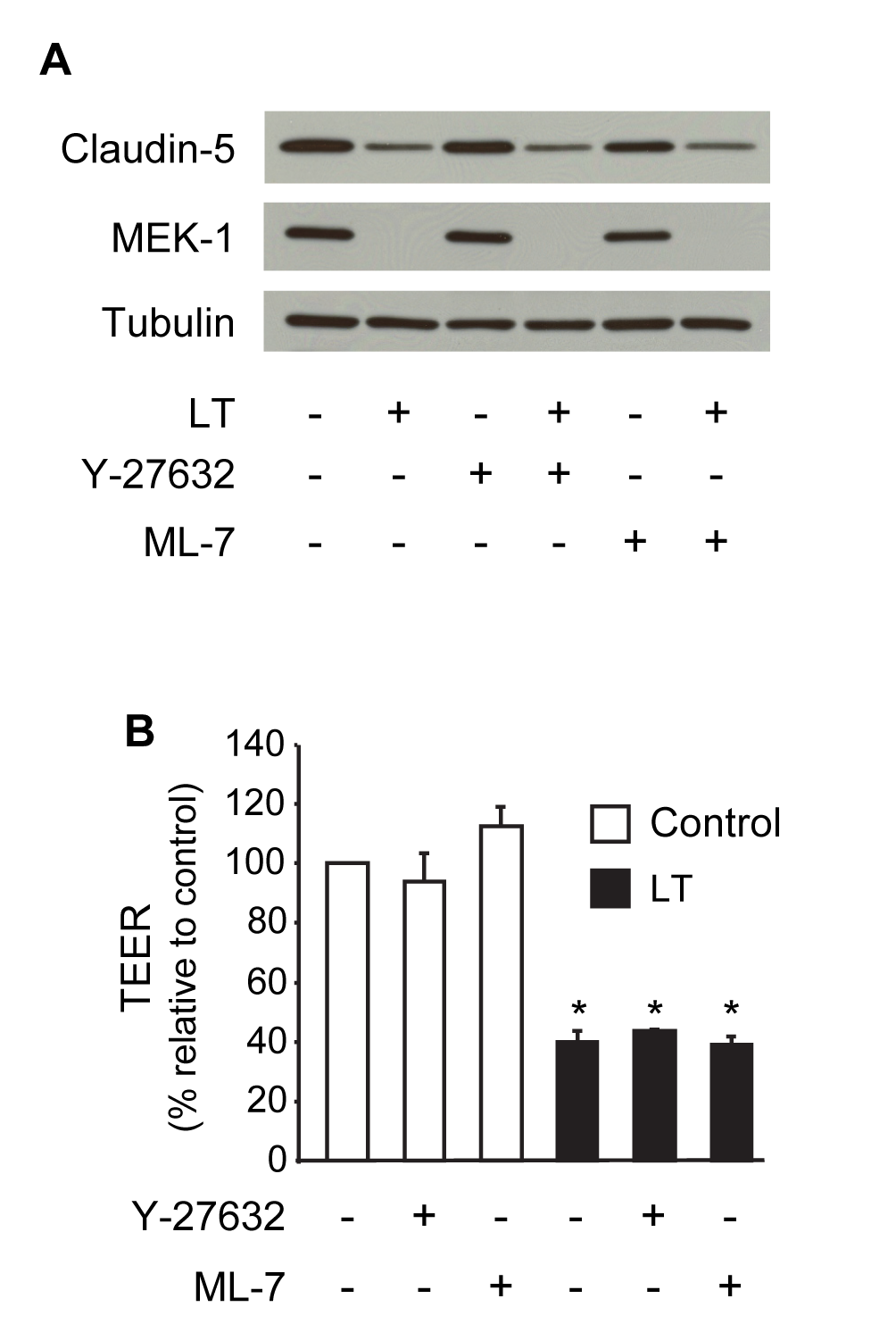

Supplement: Figure S1 — Claudin-5 downregulation is independent of actin cytoskeleton modulators, ROCK and MLCK. (A) Cells were pretreated with the ROCK inhibitor Y-27632 (5 µM) or the MLCK inhibitor ML-7 (20 µM) for 30 minutes prior to LT (100 ng/ml LF +500 ng/ml PA). Whole cell lysates were collected after 72 hours and analyzed for claudin-5 and MEK-1 by Western blot. Tubulin served as the loading control. Representative immunoblots of three separate experiments are shown. (B) Cells were grown on porous membrane inserts and pretreated with inhibitors prior to LT as indicated above. TEER readings at 72 hours were reported as the percentage of basal TEER obtained by dividing the resistance values of each treated monolayer by the resistance value of the control monolayer. The means ± SE for a minimum of three independent experiments are shown. *, p<0.05 versus control. (TIF) [file pone.0062576.s001.tif]

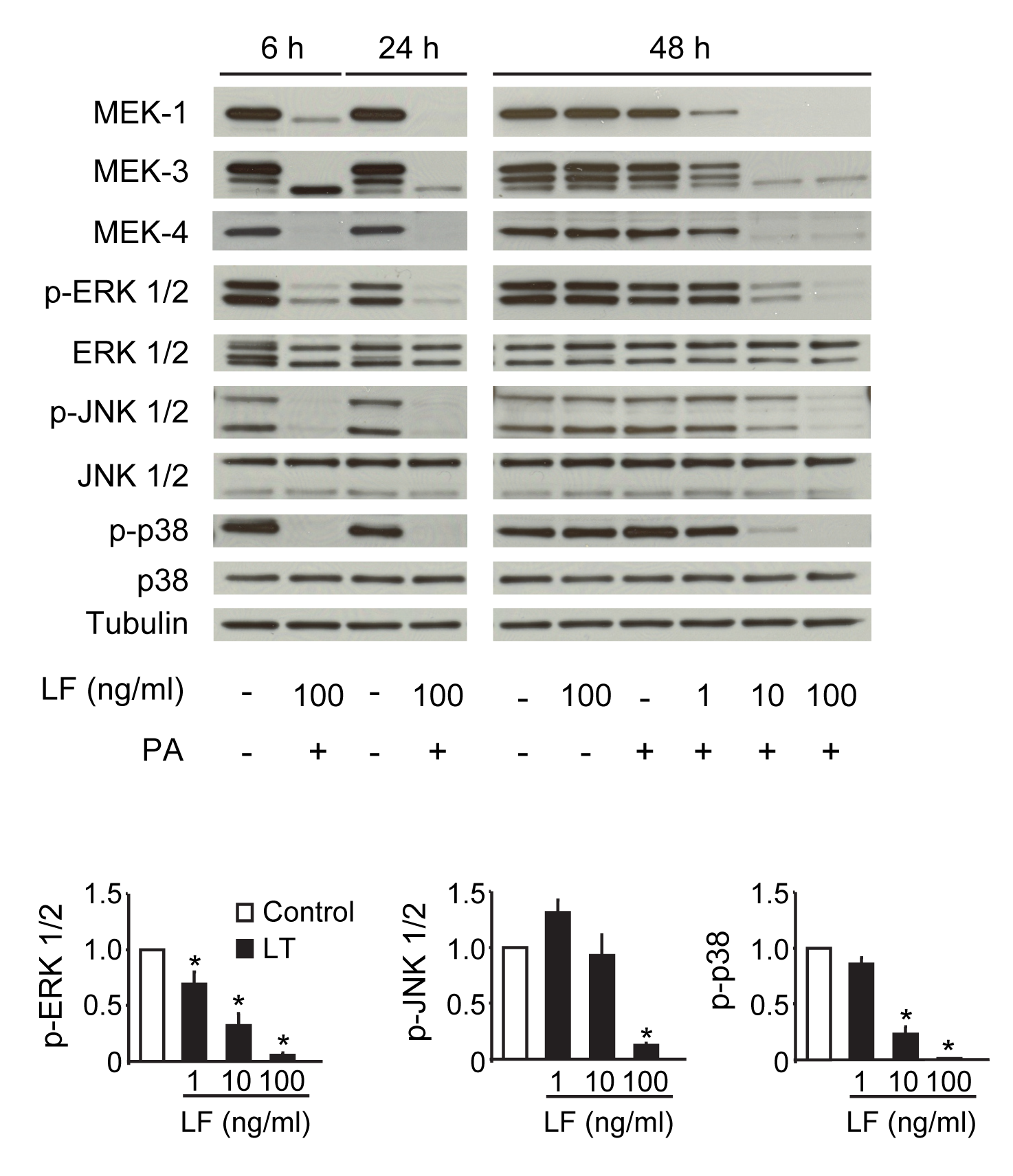

Supplement: Figure S2 — LT cleaves MEK proteins and inhibits MAPK phosphorylation. Cells were treated with medium alone, LF (100 ng/ml), PA (500 ng/ml), or the combination of PA with increasing concentrations of LF for 6, 24, and 48 hours. Whole cell lysates were analyzed by Western blot for MEK-1, MEK-3, MEK-4, and the phosphorylated and total forms of ERK 1/2, JNK 1/2, and p38. Graphs represent the densitometry analyses of phospho-ERK 1/2, JNK 1/2, and p38 as a function of LF concentration at 48 hours. Phospho-MAPK expression was normalized to tubulin and presented relative to control. Means ± SE for a minimum of three separate experiments are shown. *, p<0.05 versus control. (TIF) [file pone.0062576.s002.tif]
